# Neurodevelopment of Children Exposed to HIV But Uninfected in Zambia: Outcomes From Prospective Birth Cohort Study

**DOI:** 10.1002/jia2.70202

**Published:** 2026-09-25

**Authors:** Ethan M. Zulu, Peter C. Rockers, Leah Forman, Namwiya Musonda, Charles Nthele, Frazer Shimaingwa, Megan Bartrum, Ngosa Kawama, Carol B. Masempela, Donald M. Thea, Julie M. Herlihy

**Affiliations:** ^1^ Right to Care Zambia Lusaka Zambia; ^2^ Department of Global Health Boston University School of Public Health Boston Massachusetts USA; ^3^ Center for Health Data Science (CHDS) Boston University School of Public Health Boston Massachusetts USA; ^4^ Department of Global Health and Population Harvard T.H. Chan School of Public Health Boston Massachusetts USA; ^5^ Department of Pediatrics Boston Medical Center Boston University, Chobanian & Avedisian School of Medicine Boston Massachusetts USA

**Keywords:** cART, early childhood development, HIV, HIV‐exposed uninfected children, infants, neurodevelopment

## Abstract

**Introduction:**

Children exposed to HIV but uninfected (CHEU) number approximately 15.7 million globally. In Zambia, an estimated 56,000 CHEU are born annually, representing a substantial proportion of the national birth cohort. CHEU experience a higher risk of neurodevelopmental delay compared to their peers born HIV‐unexposed. It is unknown whether the increased neurodevelopment delay burden of CHEU has been diminished by maternal combination antiretroviral therapy.

**Methods:**

This prospective observational study analysed a subset of participants from the Zambian Infant Cohort Study (ZICS), which included 1500 pregnant women–infant dyads enrolled at a peri‐urban hospital in Zambia. Infants aged 6−24 months were enrolled in a 1:1 ratio of CHEU to CHU and were followed for 18 months. Neurodevelopment was evaluated using the Malawi Developmental Assessment Tool (MDAT) at 6‐ and 18‐month follow‐ups. Anthropometrics, Caregiver‐Reported Early Development Instruments assessments, eye‐tracking and additional assessments were conducted at 6‐, 12‐ and 18‐month follow‐ups. The primary outcomes were MDAT domain‐specific Z‐scores for gross motor, fine motor, language and social‐adaptive. A series of regression models were fitted with MDAT domain‐specific Z‐scores as dependent variables, and CHEU versus CHU status as the primary independent variable. All models were adjusted for child birth year, age at assessment, sex, mother's age, education, household size and socioeconomic status.

**Results:**

Between March 2022 and October 2024, a total of 316 mother−infant pairs were enrolled in the study (CHEU, *n* = 155; CHU, *n* = 161). MDAT assessments at the 18‐month follow‐up were done for a total of 286 infants (139 CHEU [89.7%] and 147 CHU [91.3%]). The mean age at 18‐month follow‐up was comparable between CHEU and CHU (29.1 vs. 28.0 months, respectively). Mean MDAT Z‐scores were comparable between CHEU and CHU for all domains, with adjusted mean differences in Z‐scores of 0.00 (95% CI: −0.23, 0.23) for gross motor, 0.00 (95% CI: −0.28, 0.27) for fine motor, −0.10 (95% CI: −0.48, 0.28) for language and −0.01 (95% CI: −0.32, 0.30) for social‐adaptive.

**Conclusions:**

Neurodevelopmental outcomes at endline were comparable between CHEU and CHU across all the MDAT domains in this peri‐urban Zambian cohort.

## 1 Background

Successful strategies for prevention of vertical transmission of HIV have dramatically decreased perinatal HIV acquisitions from 340,000 to 120,000 cases per year in the last 15 years [1]. Correspondingly, this has created a large and expanding number of children who are HIV‐exposed and uninfected (CHEU). As of 2024, it is estimated that there were 15.7 million CHEU globally [1, 2]. In Zambia, the population of CHEU rose from approximately 540,000 in 2014 to 680,000 in 2024, representing a 25.9% increase over the past decade [1, 3]. Multiple studies indicate that CHEU are at increased risk of neurodevelopmental delays (NDDs), including receptive and expressive language delay, fine and gross motor function differences, and autism spectrum disorder, among others [4, 5, 6, 7, 8]. By contrast, one study by Chaudhury et al. reached different conclusions, finding no significant differences in neurodevelopment at 24 months [9]. So far, existing research findings are limited due to small sample size, lack of a true HIV‐unexposed, uninfected control or were done prior to universal combination antiretroviral therapy (cART) for pregnant women, making it difficult to extrapolate to current prevention of vertical transmission guidelines. Additionally, many lack a gold standard for gestational dating, making it challenging to understand the mediating effect of prematurity, a known risk factor. Given the conflicting results to date, much uncertainty still exists about neurodevelopmental outcomes in CHEU.

The period of early childhood bears great importance for a person's quality of life throughout the lifespan, as the first years of a child's life lay the foundation for further cognitive, socioemotional, behavioural, personal, motor and language development, with a later impact on academic and professional achievement [10]. Quantifying the risk of NDDs in CHEU is critical because early intervention can be effective [11, 12]. According to the Global Burden of Disease Project, 95% of children under 5 with developmental disabilities reside in low‐ and middle‐income countries (LMICs) [13]. Therefore, understanding whether neurodevelopment is impaired in the expanding vulnerable CHEU population requires adequately powered studies to ascertain this risk.

In this study, we aimed to estimate the association between CHEU compared to children unexposed to HIV (CHU) in relation to neurodevelopmental outcomes at 2−3 years of age.

## 2 Method

### 2.1 Study Design and Participants

This prospective observational study examined a subset of participants from the Zambian Infant Cohort Study (ZICS) [14]. The parent ZICS cohort recruited pregnant women presenting to antenatal care at the hospital. Eligibility was restricted to women with ultrasound‐confirmed gestational age of less than 24 weeks at enrolment. In ZICS, gestational age was determined by a trained sonographer using a GE LOGIQ V2 ultrasound system. The system had a‐built‐in obstetric dating algorithms to estimate gestational age from foetal biometric measurements. The HIV status of the mother−infant dyad was confirmed with HIV DNA polymerase chain reaction (PCR) at antenatal, birth, 6 weeks and 6 months after birth. Information on household assets, housing characteristics and access to basic services was collected through a standardized questionnaire in the parent study. The hospital served a large, densely populated township with high levels of poverty, unemployment, food and housing insecurity, limited access to quality education and a high burden of poverty‐related health conditions. From the parent ZICS cohort, a subsample of 316 graduates was recruited for a follow‐on study with 18 months of prospective observation. Participants could enter the follow‐on study at ages 6–24 months. Enrolment criteria included: prior participation in the ZICS study, singleton birth, planning to remain in the catchment area and guardian consent.

Dyads had a study visit in accordance with the Ministry of Health (MoH) growth monitoring guidelines every 6 months. At each visit, study staff collected vaccination data, measured anthropometrics, enquired about breastfeeding, maternal mental health, diet and assessed neurodevelopment using a suite of tools described below. Data were collected at enrolment (baseline), 6 months post‐enrolment, 12 months post‐enrolment and 18 months post‐enrolment. Research nurses who were trained in questionnaire administration collected all data via direct interview of the caregiver−infant dyad at these visits. Participants were contacted via phone call or home visit to be reminded of the upcoming study visit to ensure high follow‐up rates.

### 2.2 Neurodevelopmental Assessment Tools

#### 2.2.1 Malawi Development Assessment Tool

The primary outcomes were Z‐scores in four neurodevelopmental domain measurements: gross motor, fine motor, language and social, measured at 6 months of follow‐up and endline using the Malawi Developmental Assessment Tool (MDAT) [15]. The MDAT is a context‐validated neurodevelopmental assessment tool designed for low‐resourced settings. The Z‐scores are age‐adjusted and based on the external reference population of normal children aged 0−6 from rural Malawi. Assessments were conducted in quiet rooms by two trained assessors in the preferred language of the mother or caregiver, utilizing both combined direct child observation and caregiver reporting. Once a child failed six consecutive items in a domain, the assessors proceeded to the next domain until all were completed. MDAT assessments were conducted by trained assessors who were independent of the parent cohort study (ZICS) and blinded to participants’ in‐utero HIV exposure status. In contrast, Caregiver‐Reported Early Development Instrument (CREDI), Self‐Reported Questionnaire‐20 (SRQ‐20) and anthropometrics were administered by trained research nurses involved in the parent cohort study. Although they were instructed to remain blinded to HIV exposure status, their prior involvement meant complete blinding could not be assured.

#### 2.2.2 Caregiver‐Reported Early Development Instrument

Children were assessed using the long form of the CREDI at baseline, 6, 12 and 18 months of follow‐up. The CREDI is a caregiver‐report instrument that evaluates observed age‐specific tasks for children aged 0−36 months [16]. Trained researchers administered the assessment during the specified study visits. The long form consists of 109 questions, arranged in order of increasing difficulty. The number of responses required depends on the child's age and developmental stage; each age group has a designated starting point, and the interview proceeds until five consecutive “no” answers are recorded. Scores were calculated for each subcategory (motor, cognitive, language and socioemotional) as well as for overall development.

#### 2.2.3 Self‐Reported Questionnaire‐20

The SRQ‐20 is a validated instrument to assess the mental health and wellbeing of caregivers. It is the tool of choice supported by the World Health Organization (WHO) and translated and validated in many LMICs, including Zambia [17]. It was administered by our research nurses at 6, 12 and 18 months follow‐ups.

Other secondary endpoints included Z‐scores from the diet diversity scores and Multiple Indicator Cluster Survey parent−child interaction score. Dietary diversity was assessed using a 24‐h caregiver recall based on the WHO and UNICEF Infant and Young Child Feeding guidelines.

### 2.3 Eye‐Tracking

Eye‐tracking procedures offer valuable insights into the development of cognitive, social and emotional processing during infancy [18, 19]. In this study, assessments were conducted at endline in a quiet, dimly lit room with children seated on their mother's lap approximately 60 cm from a 22‐inch monitor equipped with a Tobii Pro Fusion eye tracker (Tobii AB, Stockholm, Sweden). Three cognitive tasks were administered: the Visual Search task, which assessed the ability to locate some culturally appropriate target presented alone (one‐object condition), among identical distractors (distractor condition) or among two types of distractors (conjunction condition); the Switch‐Task, which evaluated the ability to learn and anticipate the side where a target would appear during preswitch and postswitch phases; and the Disengagement task, which measured the ability to disengage attention from a non‐face pattern or a happy or fearful face to a salient lateral stimulus. Saccadic reaction time (SRT), defined as the time taken for a participant's eyes to initiate a saccade towards a newly presented visual target, was assessed using an eye‐tracking task. Assessments were conducted by experienced research staff who had previously administered eye‐tracking procedures and received refresher training from an eye‐tracking expert from Finland before data collection to ensure standardized assessments. The assessor was blinded to the children's in‐utero HIV exposure status throughout the study. Table 1.

### 2.4 Child Growth Outcomes

Height was obtained using a portable stadiometer and weight using an electronic scale at baseline, 6 and 18 months of follow‐up. Length was measured in recumbent position for children younger than 2 years, while standing height was measured for older children. Anthropometrics were obtained by experienced research nurses who received refresher training. Children were weighed wearing light clothing. Z‐scores for height‐for‐age (HAZ), length‐for‐age (LAZ), weight‐for‐age (WAZ) and weight‐for‐length (WLZ) were calculated using the 2006 WHO child growth standards.

**TABLE 1:** Instruments used to collect data and timelines.

Instruments used to collect data	Baseline (6–24 months of age)	6 months follow‐up	12 months follow‐up	18 months follow‐up
MDAT		X		X
CREDI	X	X	X	X
SRQ‐20		X	X	X
Eye‐tracking				X
Anthropometrics	X	X		X

Abbreviations: CREDI, Caregiver‐Reported Early Development Instrument; MDAT, Malawi Development Assessment Tool; SRQ‐20, Self‐reported Questionnaire‐20.

X is indicating when the tool was used.

### 2.5 Sample Size Calculation

Assuming a conservative difference in mean MDAT domain z‐score of 0.35SD between CHEU and CHU, the proposed study was powered at 80% (two‐sided *α* = 0.05) with 129 participants per arm (258 total). To allow for anticipated loss to follow‐up, we increased the target enrolment to 175 participants per arm. This represents an allowance of approximately 26.2% loss to follow‐up.

### 2.6 Quality Assurance

Standardized developmental assessments were conducted by a child psychologist (NM) and an experienced trained examiner (NK), both blinded to participants’ HIV exposure status. Assessments of children were done in quiet clinical rooms prepared for this purpose. The child psychologist conducted 39.1% of the MDAT assessment, while the trained examiner conducted 61.9%. All the assessments done by the trained examiner were reviewed by the child psychologist. MDAT forms were reviewed daily by the study team and weekly by the study statistician. During data quality checks, the study statistician identified invalid scores, which were subsequently reviewed and corrected by the two assessors. The identified discrepancies were infrequent and limited to clerical errors, including incorrect domain score summations or data‐entry mistakes. These were verified against the original MDAT forms and corrected accordingly. Weekly meetings were held to address any concerns arising from the assessment procedures.

### 2.7 Statistical Analysis

To evaluate the association of HIV exposure status with neurodevelopmental and growth outcomes, we fitted linear regression models to estimate the mean difference between CHEU and CHU in each outcome. These models were adjusted for child age at assessment (in months), child gender, caregiver age, caregiver education and household wealth. The neurodevelopmental outcomes of interest included adjusted mean differences in MDAT scores, CREDI scores, SRT, HAZ, WAZ and WLZ growth measures. Inverse probability weighting to account for loss to follow‐up was considered during the statistical analysis in Stata version 18 but was not implemented because of high follow‐up rates in both study groups. Consequently, the small amount of missing outcome data was unlikely to meaningfully bias the study findings.

### 2.8 Ethics and Consent

The study was approved by the University of Zambia Biomedical Research Ethics Committee (FWA00000338) and the ethics committee of Boston University School of Public Health (FWA00000301). Written informed consent was obtained from all parents/guardians before enrolment.

## 3 Results

**FIGURE 1:**
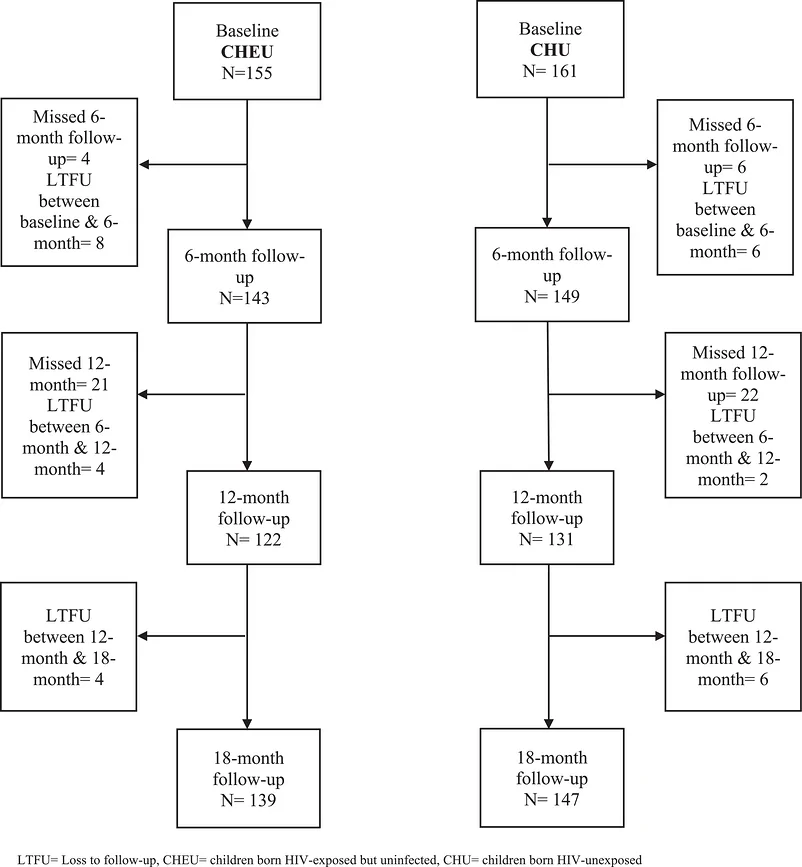
Study flow diagram.

Between March 2022 and October 2024, a total of 316 mother−infant pairs were enrolled in the study (CHEU, *n* = 155; CHU, *n* = 161). Follow‐up was available for 292 at 6 months (CHEU, *n* = 143; CHU, *n* = 149), excluding a total of 24 infants who either missed the assessment or were lost‐to‐follow up. Further follow‐up at 12 months was available for 253 children (CHEU, *n* = 122; CHU, *n* = 131). Endline assessments were performed for a total of 286 infants (139 CHEU [89.7%] and 147 CHU [91.3%]). Figure 1

Baseline characteristics of the mothers are shown in Table 2. Women living with HIV were generally older than women without HIV (30 vs. 27, *p* < 0.001). Mothers living with HIV and those without HIV exhibited similarly high unemployment rates, at 74.8% and 70.2%, respectively.

**TABLE 2:** Maternal characteristics and birth outcomes.

			Study group		
Variable	Response	Overall	CHU	CHEU	*p*‐value
Total *N*		316	161	155	—
Maternal age at ZICS enrolment (years)	*N* Mean (Std Dev)	316 29.0 (7)	161 27.0 (7)	155 30.0 (6)	< 0.001
Marital status *N* (%)	Other	46 (14.6%)	22 (13.7%)	24 (15.5%)	0.65
	Married	270 (85.4%)	139 (86.3%)	131 (84.5%)	
Employment status *N* (%)	Full‐time	61 (19.3%)	36 (22.4%)	25 (16.1%)	0.36
	Part‐time	26 (8.2%)	12 (7.5%)	14 (9.0%)	
	No	229 (72.5%)	113 (70.2%)	116 (74.8%)	
Education *N* (%)	No formal education	7 (2.2%)	4 (2.5%)	3 (1.9%)	0.030
	Primary	72 (22.8%)	30 (18.6%)	42 (27.1%)	
	Secondary	215 (68.0%)	110 (68.3%)	105 (67.7%)	
	College (and beyond)	22 (7.0%)	17 (10.6%)	5 (3.2%)	
Zambia Wealth quintile *N* (%)	First quintile	9 (2.9%)	5 (3.1%)	4 (2.6%)	0.062
	Second quintile	78 (24.9%)	36 (22.4%)	42 (27.6%)	
	Third quintile	119 (38.0%)	55 (34.2%)	64 (42.1%)	
	Fourth quintile	85 (27.2%)	48 (29.8%)	37 (24.3%)	
	Fifth quintile	22 (7.0%)	17 (10.6%)	5 (3.3%)	
Smoke during current pregnancy *N* (%)	No	314 (99.7%)	160 (100.0%)	154 (99.4%)	0.49
	Yes	1 (0.3%)	0 (0.0%)	1 (0.6%)	
Alcohol during current pregnancy *N* (%)	No	278 (88.0%)	146 (90.7%)	132 (85.2%)	0.13
	Yes	38 (12.0%)	15 (9.3%)	23 (14.8%)	
Maternal depression score—binary	< 8	304 (96.2%)	153 (95.0%)	151 (97.4%)	0.27
	> = 8	12 (3.8%)	8 (5.0%)	4 (2.6%)	
Undetectable viral load (< = 20)	No	75 (50.3%)	NA	75 (50.3%)	0.49
	Yes	74 (49.7%)	NA	74 (49.7%)	
ART initiation timing *N* (%)	HIV negative	161 (50.9%)	161 (100.0%)	NA	< 0.001
	Pre‐conception (ART > 6 months)	121 (38.3%)	NA	121 (78.1%)	
	Post‐conception (ART < 6 months)	34 (10.8%)	NA	34 (21.9%)	

Abbreviations: ART, antiretroviral treatment; CHEU, children who are HIV‐exposed but uninfected; CHU, children who are HIV‐unexposed; NA, not applicable.

Similarly, most mothers, regardless of HIV status, had not attained education beyond secondary school level. Maternal self‐report of smoking during pregnancy was very low (0.6% in mothers living with HIV and none in mothers without HIV). Of those women living with HIV, approximately half had undetectable viral load at first antenatal visit (74/149, 49.7%). A substantial proportion (121/155, 78.1%) had been on antiretroviral therapy (ART) prior to conception.

Infant sociodemographic characteristics according to maternal HIV exposure are presented in Table 3. The mean age at enrolment was comparable between the two groups (10.5 vs. 9.3). Mean ages of CHU and CHEU were similar across follow‐up visits. At 6, 12 and 18 months, mean ages were 15.4 versus 16.7 months, 22.0 versus 22.9 months, and 27.8 versus 29.1 months for CHU and CHEU, respectively. Similarly, there were no significant differences in the WAZ (−0.34 vs. −0.57, *p* = 0.075), but CHEU had a significantly lower mean LAZ than CHU at baseline (−1.35 vs. −0.92, *p* = 0.01). The food diversity score did not differ between the two groups. Exclusive breastfeeding, assessed at the 5‐month visit in the parent study, was common in both CHEU and CHU (70.3% vs. 70.2%, respectively). CHEU had similar proportions of low birth weight (<2500 g) (20.0% vs. 15.5%, *p* = 0.30) and small‐for‐gestational‐age birth (<10th percentile) (26.5% vs. 20.5%, *p* = 0.21) compared with CHU. In contrast, home stimulation, assessed by the mean number of household objects available in the home, did differ between CHEU and CHU (3.3 vs. 2.8, *p* = 0.02). Surprisingly, maternal depression was reportedly low (mothers living with HIV, 4 [2.6%], mothers living without HIV, 8 [5.0%]).

**TABLE 3:** Infant characteristics at study entry.

			Study group		
Variable	Response	Overall	CHU	CHEU	*p*‐value
Total *N*		316	161	155	—
Infant age (in months) at study enrolment	*N* Mean (Std Dev)	316 9.9 (5.8)	161 9.3 (5.0)	155 10.5 (6.5)	0.86
Sex of child	Male	171 (54.1%)	91 (56.5%)	80 (51.6%)	0.38
	Female	145 (45.9%)	70 (43.5%)	75 (48.4%)	
Height‐for‐age Z score	*N* Mean (Std Dev)	314 −1.14 (1.94)	159 −0.92 (1.97)	155 −1.35 (1.89)	0.012
Weight‐for‐age Z score	*N* Mean (Std Dev)	314 −0.45 (1.10)	159 −0.34 (0.98)	155 −0.57 (1.20)	0.075
Low birthweight	No	260 (82.3%)	136 (84.5%)	124 (80.0%)	0.30
	Yes	56 (17.7%)	25 (15.5%)	31 (20.0%)	
SGA: < 10th percentile	No	242 (76.6%)	128 (79.5%)	114 (73.5%)	0.21
	Yes	74 (23.4%)	33 (20.5%)	41 (26.5%)	
Delivery < 37 weeks—using BOE	No	286 (90.5%)	147 (91.3%)	139 (89.7%)	0.62
	Yes	30 (9.5%)	14 (8.7%)	16 (10.3%)	
Breastfeeding at 20 weeks	None^a^	16 (5.1%)	6 (3.7%)	10 (6.5%)	0.49
	Any^b^	78 (24.7%)	42 (26.1%)	36 (23.2%)	
	Exclusive	222 (70.3%)	113 (70.2%)	109 (70.3%)	
Food diversity score	*N* Mean (Std Dev)	316 3.9 (2.1)	161 3.9 (2.0)	155 3.9 (2.2)	0.88
Minimum dietary diversity (at least five out of eight categories)	No (<5 categories)	203 (64.2%)	105 (65.2%)	98 (63.2%)	0.71
	Yes (> = 5 categories)	113 (35.8%)	56 (34.8%)	57 (36.8%)	
Number of household objects available^c^	*N* Mean (Std Dev)	316 3.0 (1.4)	161 2.8 (1.3)	155 3.3 (1.5)	0.023
CREDI‐overall z‐score	*N* Mean (Std Dev)	315 −0.2 (0.7)	160 −0.2 (0.7)	155 −0.2 (0.7)	0.86

Abbreviations: BOE, best obstetric estimation; CHEU, children who are HIV‐exposed but uninfected; CHU, children who are HIV‐unexposed; SGA, small for gestation age.

^a^ Never breastfed.

^b^ Mixed feeding.

^c^ Measured using CREDI tool.

Coefficients are the estimated CHEU–CHU difference (CHU is the reference group). Means are unadjusted group means. Unadjusted models regress each outcome on CHEU status only. Adjusted models control for child age at the time of assessment, child date of birth and sex; mother's age and education; and household size and wealth quintile.

### 3.1 MDAT Outcomes

Neurodevelopmental outcomes for CHEU and CHU are presented in Table 4. At 18‐month follow‐up, using a regression model adjusted for child date of birth, age at assessment, sex, mother's age, education, household size and wealth showed comparable MDAT Z‐scores between CHEU and CHU across all four domains. Mean MDAT Z‐scores were very similar for gross motor domains (1.11 vs. 1.19), fine motor (1.17 vs. 1.14), language (CHEU: 1.14 vs. CHU: 1.28) and social‐adaptive domains (0.03 vs. 0.02). After adjustment for potential confounders, there was no difference in adjusted mean Z‐scores for any of the MDAT domains. Mean SRT measured at the 18‐month follow‐up using the eye‐tracking device was similar between CHEU and CHU (321.4 vs. 318.1, 95% CI: −6.81 to 16.81).

**TABLE 4:** Relationship between CHEU status and child development.

Assessment time point	Mean		Unadjusted			Adjusted for age at assessment		
Outcome	CHU	CHEU	Mean difference	(95% CI)	*p*	Adjusted mean difference	(95% CI)	*p*
**MDAT—Gross motor z‐score**								
6 months	0.31	0.34	0.04	(−0.27, 0.35)	0.82	−0.07	(−0.38, 0.23)	0.64
18 months	1.19	1.11	−0.08	(−0.31, 0.15)	0.50	0.00	(−0.23, 0.23)	1.00
**MDAT—Fine motor z‐score**								
6 months	0.47	0.49	0.01	(−0.31, 0.34)	0.93	−0.11	(−0.45, 0.23)	0.53
18 months	1.14	1.17	0.02	(−0.25, 0.29)	0.86	−0.00	(−0.28, 0.27)	0.97
**MDAT—Language z‐score**								
6 months	0.43	0.70	0.27	(0.02, 0.53)	0.036	0.15	(−0.11, 0.42)	0.26
18 months	1.28	1.14	−0.13	(−0.50, 0.23)	0.47	−0.10	(−0.48, 0.28)	0.61
**MDAT—Social‐adaptive z‐score**								
6 months	0.28	0.26	−0.01	(−0.31, 0.28)	0.93	−0.02	(−0.32, 0.28)	0.90
18 months	0.02	0.03	0.01	(−0.29, 0.30)	0.97	−0.01	(−0.32, 0.30)	0.95
**CREDI—Cognitive z‐score**								
6 months	−0.92	−0.89	0.03	(−0.17, 0.22)	0.78	0.01	(−0.20, 0.21)	0.95
12 months	−0.15	−0.05	0.10	(−0.15, 0.35)	0.43	0.01	(−0.24, 0.25)	0.96
18 months	0.27	0.22	−0.05	(−0.28, 0.18)	0.69	−0.05	(−0.29, 0.19)	0.69
**CREDI—Language z‐score**								
6 months	−0.95	−0.93	0.01	(−0.22, 0.25)	0.91	−0.03	(−0.27, 0.20)	0.77
12 months	−0.10	0.06	0.17	(−0.06, 0.39)	0.14	0.11	(−0.11, 0.32)	0.32
18 months	−0.09	−0.14	−0.04	(−0.23, 0.14)	0.64	0.00	(−0.18, 0.19)	0.98
**CREDI—Motor z‐score**								
6 months	−0.73	−0.76	−0.03	(−0.22, 0.17)	0.79	−0.04	(−0.25, 0.16)	0.68
12 months	−0.11	−0.07	0.04	(−0.24, 0.32)	0.78	−0.04	(−0.32, 0.23)	0.75
18 months	0.16	0.13	−0.03	(−0.28, 0.22)	0.80	−0.05	(−0.32, 0.21)	0.69
**CREDI—Social‐emotional z‐score**								
6 months	−0.69	−0.62	0.06	(−0.15, 0.28)	0.57	0.03	(−0.19, 0.26)	0.78
12 months	0.10	0.17	0.07	(−0.17, 0.32)	0.57	−0.02	(−0.26, 0.22)	0.90
18 months	0.55	0.54	−0.02	(−0.24, 0.21)	0.88	−0.03	(−0.27, 0.20)	0.77
**HAZ**								
6 months	−0.89	−1.20	−0.31	(−0.81, 0.19)	0.22	−0.32	(−0.85, 0.20)	0.23
18 months	−1.64	−1.89	−0.25	(−0.57, 0.08)	0.14	−0.17	(−0.50, 0.15)	0.30
**WAZ**								
6 months	−0.60	−0.68	−0.08	(−0.35, 0.19)	0.58	−0.07	(−0.35, 0.21)	0.63
18 months	−0.67	−0.79	−0.12	(−0.38, 0.14)	0.37	−0.13	(−0.40, 0.14)	0.34
**WHZ**								
6 months	−0.38	−0.06	0.32	(−0.08, 0.71)	0.12	0.32	(−0.09, 0.72)	0.12
18 months	0.39	0.33	−0.06	(−0.32, 0.20)	0.66	−0.12	(−0.40, 0.15)	0.38
**SRT** ^a^ **(ms)**								
18 months	318.05	321.42	3.37	(−8.39, 15.14)	0.57	5.00	(−6.81, 16.81)	0.41

^a^ Saccadic reaction time.

### 3.2 CREDI Outcomes

Mean CREDI Z‐scores at endline were comparable between CHEU and CHU across all developmental domains. The adjusted mean Z‐scores were similar for cognition (0.22 vs. 0.27), language (−0.14 vs. −0.09), motor (0.13 vs. 0.16) and social‐emotional development (0.54 vs. 0.55), providing little evidence of differences associated with in utero HIV exposure (all *p*>0.05).

### 3.3 Growth Outcomes

Anthropometrics were assessed as secondary outcomes at 6 and 18 months of follow‐up. Mean growth Z‐scores were similar between CHEU and CHU at both time points. Mean HAZ declined in both groups between 6 and 18 months of follow‐up, with CHEU having mean scores of −1.20 and −1.89, and CHU having mean scores of −0.89 and −1.64, respectively. WAZ remained relatively stable over time, changing by less than 0.15 z‐score units, while WHZ were comparable between groups at both assessments.

## 4 Discussion

While the global CHEU population continues to grow, evidence comparing neurodevelopmental outcomes between CHEU and CHU remains heterogeneous [5]. In our cohort, we found no clinically meaningful differences in neurodevelopmental outcomes for ages 2−3 years between CHEU and CHU. The two groups had very similar mean MDAT Z‐scores across all four domains, with no indication of a difference in neurodevelopmental functioning. These findings are consistent with Struyf et al., who reported similar cognitive development among CHEU and CHU in Malawi [20]. Our findings offer some reassurance that HIV exposure alone may not substantially impact neurodevelopment in children in Zambia. However, the study was powered to detect a difference in the range of 0.3−0.35 standard deviation and smaller true differences may not have been detected. Interestingly, mean Z‐scores for both CHEU and CHU exceeded 1.0 in the gross motor, fine motor and language domains at endline, indicating performance above the MDAT reference population. This may reflect differences in socioeconomic status, healthcare access or indeed geographical context between our peri‐urban cohort and the Malawian population used for MDAT standardization.

The Zambia Infant Cohort demonstrated an increase in preterm birth among CHEU, particularly in cases where cART was initiated after conception [21]. However, this same cohort did not demonstrate a significant difference in infant hospitalizations or death among CHEU. The prevalence of preterm birth was similar between CHEU and CHU. However, the small number of preterm births limited subgroup analyses and prevented robust conclusions regarding its association with neurodevelopmental outcomes.

Various tools are used to assess child development. We used the CREDI, which rely on parental report to assess the motor, cognitive and socioemotional skills of the children. The CREDI is less likely to be biased against children who are unfamiliar with clinical assessments or shy with strangers [22]. In our study, after controlling for child date of birth, age at assessment, sex, maternal age, education, household size and wealth, mean CREDI Z‐scores at endline were comparable between CHEU and CHU across all developmental domains, providing little evidence of differences associated with in‐utero HIV exposure. Similar findings have been reported in other studies, which also observed modest, non‐significant differences in neurodevelopmental outcomes between CHEU and CHU. For example, Chaudhury et al. found that CHEU children performed comparably to CHU children on neurodevelopmental assessments conducted at 24 months of age [9].

CHEU remain at a heightened risk of stunting and wasting [23, 24]. One of the key drivers of this vulnerability is their disproportionate exposure to food insecurity [25]. Households affected by HIV often face persistent nutritional and economic challenges, which further undermine children's growth, development and academic performance. Although the primary objective of this study was not to examine the determinants of malnutrition, anthropometric outcomes were measured and revealed that both CHEU and CHU were found to be at risk of stunting and wasting according to the 2006 WHO child growth standards [26]. These findings align with an earlier study by Nyemba et al., which pooled data from Zambia and South Africa [27, 28]. Given these risks, vigilant growth monitoring and targeted nutritional support should be prioritized for both CHEU and CHU, particularly those living in impoverished communities. Failure to do so may contribute to diminished educational outcomes and increase the likelihood of intergenerational impacts on health and human capital.

This study had several limitations. First, eye‐tracking data were collected only at endline, which limited their use in the current analyses; however, these data may be valuable for future exploratory studies. Second, residual confounding remains a concern in observational research. However, we sought to minimize this by identifying and adjusting for key confounders a priori during both study design and analysis, as well as blinding of MDAT assessors. Third, because contextual factors such as the effectiveness of programmes for prevention of vertical transmission of HIV strongly influence neurodevelopmental health outcomes, the single‐site nature of our study limits the generalizability of the findings. Fourth, repeated measures analyses could have provided insights into potential subgroups of children with persistently lower or declining developmental trajectories. However, these were beyond the scope of the study due to resource limitations.

Despite these limitations, the study had several strengths. We collected robust longitudinal data on mother–infant pairs from the first antenatal visit, gold‐standard gestational dating via ultrasound, to approximately 3 years of age. The CHEU and CHU groups were well characterized and recruited from the same community, reducing heterogeneity between comparison groups. Follow‐up rates were high throughout the study period. Additionally, the cohort demonstrated high levels of maternal viral suppression and breastfeeding, indicating the effectiveness of the vertical transmission prevention programme and representing an optimal context for preventing vertical HIV transmission.

## 5 Conclusions

In conclusion, our findings on endline neurodevelopmental outcomes among CHEU compared with CHU in a peri‐urban Zambian setting are encouraging. CHEU performed comparably to CHU across language, gross motor, fine motor and social–adaptive domains. However, as the study was powered to detect a difference in the range of 0.3−0.35 SD, larger and longer‐term cohort studies are needed to determine whether the subtle, non‐significant differences observed in language and motor development translate into clinically meaningful disparities over time.

Continued developmental surveillance of CHEU throughout childhood remains essential, given their documented increased vulnerability to neurocognitive and psychiatric risks in middle childhood. Although we found no statistically significant differences in HAZ, WAZ or WHZ between CHEU and CHU, both cohorts had mean HAZ and WAZ scores well below the WHO population average, highlighting the broader burden of undernutrition in this vulnerable population. Holistic early childhood development interventions will, therefore, be required to address the multifactorial determinants of NDD among children living in impoverished communities with limited access to quality health and social services.

## Author Contributions

EMZ contributed to study design, analysis and drafting the article; PCR substantially contributed to conception, design, analysis, interpretation and reviewing the article; LF contributed to data analysis and reviewing the article; NM contributed to method design and data acquisition; MB contributed to design and data acquisition; NK, CN, FS and CBM contributed to data acquisition; DMT contributed to conception, design and internal review; JMH contributed to conception, design, data interpretation and internal review.

## Conflicts of Interest

The authors declare that they have no conflicts of interest.

## Data Availability

Data that support the results of this study are available on the BEDAC repository and can be shared upon request.
